# Thoughts and beliefs of healthcare workers regarding the coronavirus disease 2019 (COVID-19) vaccine and which messaging themes might affect vaccine confidence and recommendation of the vaccine

**DOI:** 10.1017/ash.2022.50

**Published:** 2022-05-04

**Authors:** S. Shaefer Spires, Jenna L. Clark, Rebecca Rayburn-Reeves, Elizabeth Dodds Ashley, Avani Desai, Jan Lindemans

**Affiliations:** 1 Duke Center for Antimicrobial Stewardship and Infection Prevention, Duke University School of Medicine, Durham, North Carolina; 2 Center for Advanced Hindsight, Social Science Research Institute, Duke University, Durham, North Carolina

## Abstract

We surveyed healthcare workers within the Duke Antimicrobial Stewardship Outreach Network (DASON) to describe beliefs regarding coronavirus disease 2019 (COVID-19) vaccination and their decision-making process behind vaccination recommendations. In contrast to the type of messaging that appealed most on a personal level to the healthcare workers, they preferred a more generic message emphasizing safety and efficacy when making vaccination recommendations.

The current availability of 3 vaccines for COVID-19 in the United States is a remarkable scientific feat. Despite their proven efficacy and safety, these vaccines are no exception to the problem of vaccine hesitancy.^1–3^

Healthcare worker (HCW) interactions with patients play a key role in vaccine confidence.^4–7^ As professionals in healthcare, trained to evaluate science with rational arguments, often our response to vaccine hesitancy is to repeat the evidence.^8^ For example, an emotionally evocative news story about a nurse developing anaphylaxis after vaccination would typically be countered by describing the low rates of vaccine side effects and the greater risk of illness from infection. Clearly this rational approach has missed the mark for a substantial proportion of people.^3^

In contrast, behavioral science theorizes that emotions and core beliefs play an essential role in health decisions, whereas interventions for vaccination hesitancy might rely on appeals to anticipated regret, fear of disease, or even social norms. Using behavioral science methods, we sought to better understand HCW beliefs about the COVID-19 vaccine in our network of hospitals and among our colleagues. We then sought to determine what messaging themes are most effective at increasing both HCW likelihood of vaccination against COVID-19 and vaccination recommendations to their patients.

## Methods

The Duke Antimicrobial Stewardship Outreach Network (DASON) partnered with the Center for Advanced Hindsight (CAH) to assess HCW experience and beliefs around COVID-19 and the perceived efficacy of messaging themes aimed at increasing COVID-19 vaccination. An online survey was e-mailed through 8 hospital listservs in DASON as well as through the North Carolina Medical Society during the month of February 2021. We randomly assigned participants to see 1 of 3 thematic messages about COVID-19 vaccines or to a no-message control group:

1. Process safety: Rapid development and testing of COVID-19 vaccines were made possible through a combined effort worldwide and vaccines are both safe and effective.
2. Appeal to normalcy: What do you miss about a prepandemic life? How much would you pay to have it back? For life to return to normal, vaccination is the key.
3. Risk assessment: Decisions involving uncertain outcomes create 2 ways to be wrong—What is the risk of vaccinating versus the risk of COVID-19?

Participants were then asked a series of questions regarding their perception of the passage (eg, helpful, believable) as well as their likelihood of sharing it with their patients. We used the χ^2^ test to compare willingness to share the passage by condition and linear regression to test passage ratings by condition. This study was approved by the Institutional Review Board of Duke University (protocol no. 2021-0286). Full survey available online.^9^

## Results

Our final sample included 674 survey respondents, almost exclusively from North Carolina (98%) (Table 1). The sample comprised 84% women and 83.3% were of White race. The most common role represented was nursing (34%), and 72% of the respondents came into direct contact with COVID-19 patients. The largest portion of respondents worked in a hospital with bed capacity ranging from 100 to 199 beds.

**Table 1.** Characteristics of Survey Respondents

Characteristic	Total
Total sample, no.	890
Excluded due to incomplete or duration to complete survey <2 min, no.	216
Total included in analysis, no.	674
**Location, %**	
North Carolina	98
Virginia, New York, Delaware, and North Dakota	2
**Sex, %**	
Female	84
Male	15
Nonbinary/Gender queer or other	<1
**Ethnicity, %**	
White	85
Black/African American	7
Multiracial	3
Native American	2
Latinx	1
South Asian	1
East Asian, Southeast Islander, Middle Eastern or North African	<1
**Political ideology, %**	
Republican	43
Independent	25
Democrat	17
No preference	13
Other	2
**Job role, %**	
Nurse	34
Other direct patient service provider (eg, respiratory therapist or radiology technician)	31
Indirect patient service provider (eg. nutrition service)	14
Administrator	8
Nurse assistant	5
Physician/Midlevel provider	5
Pharmacist	3
Direct contact with COVID-19 patients	72
**Hospital bed size**	
<100	8
100–199	44
200–299	18
300–399	14
400–499	8
500+	8

Of the 98% of respondents who had been offered, 80% had already accepted the COVID-19 vaccine. Of the smaller cohort who had not received the vaccine (n = 127), the top 3 reasons for hesitancy dealt with safety concerns, such as “want to ensure the vaccine is safe,” “the vaccine was created too quickly,” and “wait to see the vaccine’s effectiveness.” Vaccine acceptance rates also differed by reported political affiliation, with Democrats showing the highest uptake (90%), followed by Independents (80%), and finally Republicans (75%). Democrats were also more likely to share the message they read to their patients (94%) compared with 87% of Republicans. Among those who had not been vaccinated, we did not detect a significant difference across messaging interventions regarding vaccine acceptance (all *P* values >.13).

**Fig. 1.**
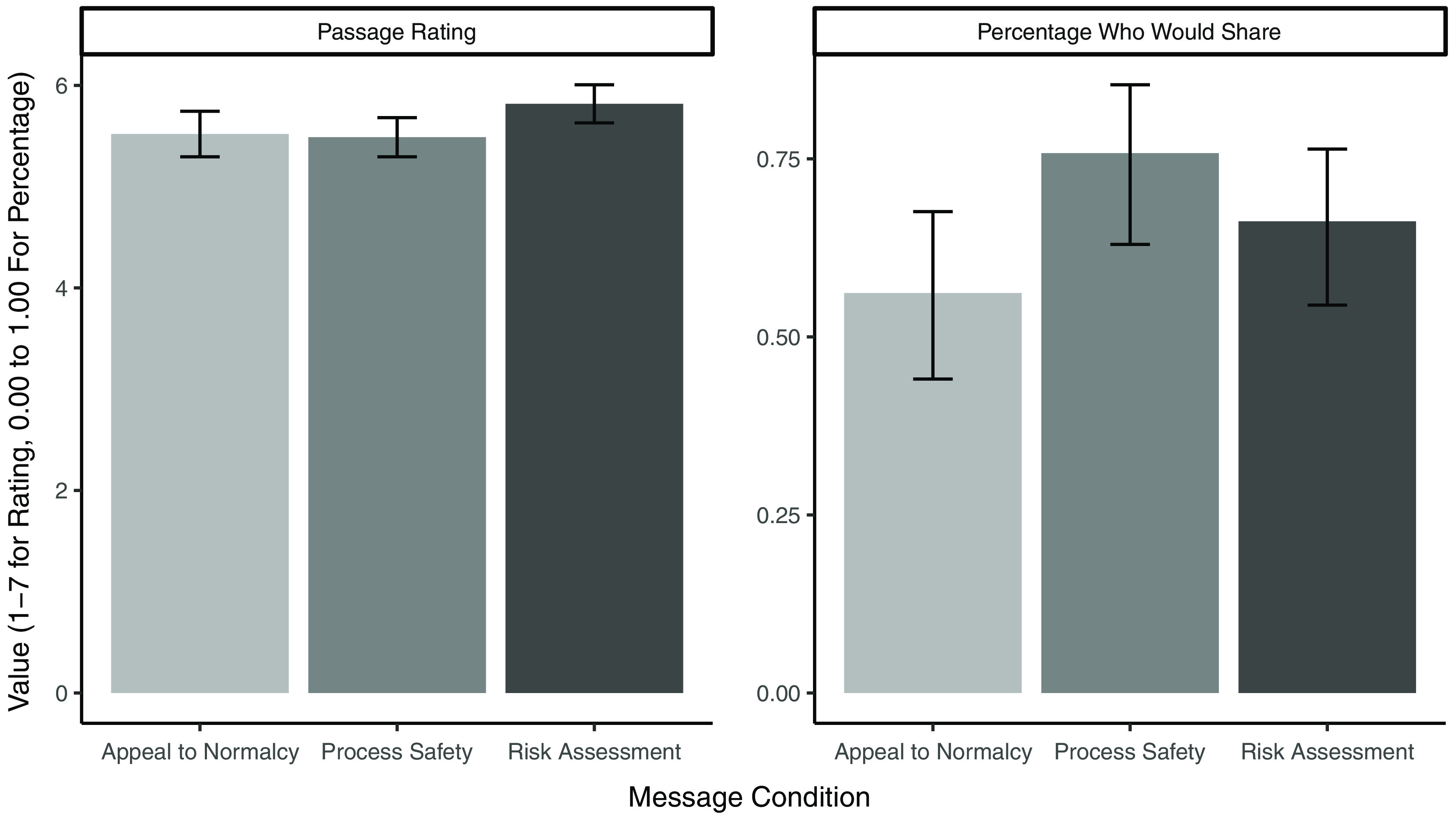
Passage rating and willingness to share by message condition. *Note:* Error bars indicate 95% confidence intervals.

Generally, HCWs were very comfortable recommending the COVID-19 vaccine to patients, as indicated by an average rating of 6.33 on a scale from 1 to 7 (1 = extremely unlikely to 7 = extremely likely). However, participants found the risk assessment message to be the most trustworthy, believable, and correct on a scale of 1–7 (1 = strongly disagree to 7 = strongly agree). When these 3 ratings are combined, the risk assessment message is rated significantly higher than either the appeal to normalcy message (*P* = .04) or the process safety message (*P* = .03) (Fig. 1). However, those assigned to the process safety message were more likely to say they would share their message with patients (75.8%) than those assigned to the normalcy condition (56.2%; *P* = .09), and the risk assessment condition fell between these 2 conditions (66.2%).

## Discussion

Fortunately, HCWs generally had high COVID-19 vaccine acceptance rates and were likely to recommend the vaccine to patients. Regarding those who were hesitant, there was no difference across messages on increasing their acceptance, but the small size of the cohort makes it difficult to draw any conclusions.

Although HCWs generally gave all 3 passages high ratings, the risk assessment message had the highest ratings as trustworthy, believable, and correct; however, the process safety message ranked higher when asked whether they would share this passage with a patient. This finding suggests rhetoric that appeals on a personal level is not necessarily what HCWs would recommend to their patients. In other words, an HCW may prefer a message that feels credible to them over one that they feel is more likely to change patient behavior.

The qualitative data we gathered provides some insight into this difference. HCWs considered managing vaccine discussions in both ideological and practical terms. On an ideological level, patient autonomy was cited as an issue: HCWs “want to educate without adding opinions.” In a practical sense, HCWs were concerned with avoiding patient conflict, and also about their own ability to discuss this new technology: Vaccines are “not in my scope of practice”; I’m “not sure if I can inform clearly”; and “I’m not knowledgeable enough to recommend the vaccine to others.” These beliefs may explain the appeal of a process safety message because it was succinct and easy to memorize and was very similar to public health messaging at that time. In contrast, the risk assessment message requires one to understand certain statistical facts and discuss relative risk in order to share. When there is novel health technology we are tasked to endorse, bullet points are an efficient way to learn facts to discuss and create a familiar tone as they are reiterated.

This study had several limitations. These results may not be generalizable across space or time because the study captured opinions from February 2021 among HCWs concentrated in North Carolina. However, our findings suggest that HCWs are as likely to show similar concerns about vaccine safety as those captured in the general population. Additionally, our finding that HCWs may prefer to share a message they find credible over one they find persuasive suggests that the rational model of thinking may not even apply to us, let alone our patients. Physicians and other professionals should consider adopting methods from behavioral science to determine the true reasons why patients fail to comply with directions or take advantage of preventative services. Understanding human behavior is the first step to changing it.

## Supplementary material

For supplementary material accompanying this paper visit https://doi.org/10.1017/ash.2022.50. click here to view supplementary material

## References

[1] Polack FP , Thomas SJ , Kitchin N , et al. Safety and efficacy of the BNT162b2 mRNA COVID-19 vaccine. N Engl J Med 2020;383:2603–2615. 33301246 10.1056/NEJMoa2034577 PMC7745181

[2] Barda N , Dagan N , Ben-Shlomo Y , et al. Safety of the BNT162b2 mRNA COVID-19 vaccine in a nationwide setting. N Engl J Med 2021;385:1078–1090. 34432976 10.1056/NEJMoa2110475 PMC8427535

[3] Intent to get a COVID-19 vaccine rises to 60% as confidence in research and development process increases. Pew Research Center Science & Society website. https://www.pewresearch.org/science/2020/12/03/intent-to-get-a-covid-19-vaccine-rises-to-60-as-confidence-in-research-and-development-process-increases/. Published 2020. Accessed April 10, 2022.

[4] Pandolfi E , Marino MG , Carloni E , et al. The effect of physician’s recommendation on seasonal influenza immunization in children with chronic diseases. BMC Public Health 2012;12:984. 23153092 10.1186/1471-2458-12-984 PMC3585468

[5] Wiley KE , Leask J. Respiratory vaccine uptake during pregnancy. Lancet Respir Med 2013;1:9–11. 24321792 10.1016/S2213-2600(13)70024-9

[6] Reiter PL , Gilkey MB , Brewer NT. HPV vaccination among adolescent males: results from the National Immunization Survey–Teen. Vaccine 2013;31:2816–2821. 23602667 10.1016/j.vaccine.2013.04.010 PMC3672374

[7] Brewer NT , Chapman GB , Rothman AJ , Leask J , Kempe A. Increasing vaccination: putting psychological science into action. Psychol Sci Public Interest 2017;18:149–207. 29611455 10.1177/1529100618760521

[8] Hornsey MJ , Fielding KS. Attitude roots and Jiu Jitsu persuasion: understanding and overcoming the motivated rejection of science. Am Psychol 2017;72:459. 28726454 10.1037/a0040437

[9] Rayburn-Reeves RM , Lindemans JW , Clark J , et al. COVID-19 hesitancy in healthcare workers—Feb 2021, OSF. https://osf.io/5z9he/. Published November 1, 2021. Accessed March 11, 2022.

