# Supplementary material for: Thoughts and beliefs of healthcare workers regarding the coronavirus disease 2019 (COVID-19) vaccine and which messaging themes might affect vaccine confidence and recommendation of the vaccine
Source: Antimicrob Steward Healthc Epidemiol. 2022 May 4;2(1):e76. doi: 10.1017/ash.2022.50 (PMC9726571; doi:10.1017/ash.2022.50)
Supplement: Supplementary file 1 [file S2732494X2200050Xsup001.pdf]

Consent

Protocol # 2021-0286

Key Information
Researchers from the Center for Advanced Hindsight at Duke University and the Duke Center for Antimicrobial Stewardship and Infection Prevention are conducting a research study to examine how to make our response to the COVID-19 pandemic more effective.

You must be at least 18 years of age and be currently employed by a healthcare facility to participate in this study. If you decide to participate, you will complete a 10-minute survey to assess possible messaging campaigns around the COVID-19 vaccine. The survey will also ask you questions about COVID-19 and about you, like your age, race, and political affiliation.

In return for your participation, you may opt to enter a drawing to win 1 of 5 gift packs containing a water bottle, face mask, and light fleece jacket with the Duke Center for Antimicrobial Stewardship and Infection Prevention's petri dish logo. If you opt to enter the drawing, you will need to provide your email address. You may participate in the study without providing your email address, but you will not be entered into the drawing.

Your email address will be stored separately from the survey data and will be used only for contacting drawing winners. If you win the drawing, CAH researchers will contact you for your name and mailing address to ship your prize pack. Your name, email address and mailing address will not be linked to your survey responses and will be deleted after all gift packs have been distributed. Survey data will be stored in a secure database at Duke University. CAH may use the data for future research and publication, or share results with collaborators, but those data will not contain any identifying information about

you.

Your participation is entirely voluntary. You may skip any question or withdraw from the study at any time. If you have any questions about the survey or your participation, email jenna.clark@duke.edu. If you have questions about your rights as a research participant, contact the Duke research review committee at (919) 684-3030 or campusirb@duke.edu and reference protocol ID# 2021-0286.

Do you agree to participate in the study?

I agree

I do not agree

How old are you (in years)?

Are you a licensed medical provider?

Yes

No

Do you work in a hospital, clinic, or other health care facility?

Yes

No

I'm sorry, but you do not qualify for our study. Thank you for your interest!

Hypothetical Scenarios - Process

Thank you for agreeing to participate in our study! We would like you to take a look at a public health campaign about vaccination, and give your opinion about it. Please read the passage closely and carefully.

Some people are afraid to take a COVID-19 vaccine because of how quickly the vaccine has been developed. But actually, there's no reason to be afraid. Why?

The vaccine may have been developed quickly, but that doesn't mean any corners were cut in testing it for safety.

Vaccines have to go through an extensive series of clinical trials to prove they're both safe and effective. This process has worked for countless vaccines over the years, and it was carried out for COVID-19 vaccines in just the same way. Over 43,000 people had already been involved in testing Pfizer's vaccine by mid-November, and another 30,000 have helped test Moderna's. Among all 70,000+ people, the vaccine didn't cause a single serious side effect. Small numbers of people felt minor side effects, like headaches or fevers, but they recovered quickly in a day or two.

So anyone who gets a vaccine, even in the first phase, shouldn't think of it as being a guinea pig - just first in line for the new wider rollout.

Hypothetical Scenarios - SDT

Thank you for agreeing to participate in our study! We would like you to take a look at a public health campaign about vaccination, and give your opinion about it. Please read the passage closely and carefully.

Nearly all of our decisions involve some degree of uncertainty. When faced with the decision about whether to get a vaccine for the COVID-19 virus, there are two ways you could make a mistake.

Let’s say you decide not to get vaccinated because you don’t think you will get the virus/the virus won't make you sick.

- If you're right, it would mean you correctly assumed you weren't at risk and didn't have to get the vaccine.

- **If you're wrong, you catch the virus and risk getting yourself and your loved ones very sick.**

Now let's say that you decide to get vaccinated because you think you will be at risk for catching or getting sick from the virus.

- If you're right, then you would have caught the virus if you hadn't been vaccinated and you've saved yourself and your loved ones from the virus.
- **If you're wrong, you took a vaccine but did not need it and there is a small chance you will experience symptoms like a mild cold.**

Not Vaccinating = HIGH RISK of illness | Vaccinating = LOW RISK of illness

Which risk would you rather take?

Hypothetical Scenarios - Appeal of Normalcy

Thank you for agreeing to participate in our study! We would like you to take a look at a public health campaign about vaccination, and give your opinion about it. Please read the passage closely and carefully.

Take a moment to tell us a few things about your life or routine before the COVID-19 pandemic that you miss. For example, you might say "Spending time with my friends."

I miss...	
I miss...	
I miss...	

Many people's lives will never go back to normal after the pandemic, but there are still things about our old lives that we can hope to enjoy again someday.

Imagine a hypothetical scenario where you could simply pay to have your old hobbies, activities, and routines back. How much would you be willing to pay (in dollars?)

This scenario isn't entirely hypothetical. Professor Ugur Sahin, the co-creator of the Pfizer COVID-19 vaccine, has said that he thinks life could be back to normal next year - IF everyone receives their vaccinations by autumn 2021. It won't be enough to just hope other people choose to get their shots. If we want to safely see our loved ones, eat in restaurants without worry, and travel freely again, we'll all have to do our part.

This means that a return to normalcy is in your hands after all, and you don't have to pay your entire life savings for it. Instead, all you have to do is get two little shots.

Passage-Specific Outcomes

Please let us know what you think about the passage we've just shared with you.

This passage was _______.

	Strongly agree	Agree	Somewhat agree	Neither agree nor disagree	Somewhat disagree	Disagree	[illegible]
Understandable/clear	○	○	○	○	○	○	
Helpful	○	○	○	○	○	○	
Not something I had thought about before	○	○	○	○	○	○	
Correct	○	○	○	○	○	○	
Believable	○	○	○	○	○	○	
Trustworthy	○	○	○	○	○	○	
Offensive	○	○	○	○	○	○	

Do you think the passage you just read would help your patients feel more comfortable about getting the vaccine?

Definitely yes	Probably yes	Might or might not	Probably not	Definitely not

Would you share this passage with your patients (e.g., by handing out a flyer, hanging a poster, or talking to a patient directly?)

Yes

Maybe

No

Do you have any other thoughts about the passage that you would like to share?

COVID-19 Vaccination Outcomes

An FDA-authorized vaccine to prevent COVID-19 is currently being distributed to hospitals nationwide. Has a COVID-19 vaccine already been offered to you?

Yes

No

I don't know

Did you accept the COVID-19 vaccine? (If you have only received the first dose of two, say 'Yes').

Yes	No, but I probably will later	No, but I might later	No, and I probably won't later	No, and I definitely won't

Imagine that the COVID-19 vaccine will be offered to you next week free of charge in a very safe location nearby. How likely are you to accept the vaccine?

Not at all likely	Slightly likely	Somewhat likely	Pretty likely	Very likely

How much do you agree with the following statements?

	Strongly disagree	Disagree	Slightly disagree	Neither agree nor disagree	Slightly agree	Agree	Strongly agree
I would do almost anything to get my normal life back.	○	○	○	○	○	○	○
Vaccination will help me get my normal life back.	○	○	○	○	○	○	○
Getting my normal life back isn't possible.	○	○	○	○	○	○	○

Now we'd like you to rate how you feel about certain people on a *feeling thermometer* using a scale of 0 to 100. The **higher** the number, the **warmer** or more favorable you feel toward that person; the **lower** the number, the **colder** or less favorable you feel. You can pick any number between 0 and 100.

	Very cold/unfavorable				No feeling at all				Very warm/favorable	
	0	10	20	30	40	50	60	70	80	90	100
Health care workers who choose to get vaccinated against COVID-19											
Health care workers who choose NOT to get vaccinated against COVID-19											
My friends and family who choose to get vaccinated against COVID-19											

	Very cold/unfavorable	No feeling at all	Very warm/favorable

	0	10	20	30	40	50	60	70	80	90	100
My friends and family who choose NOT to get vaccinated against COVID-19											

Which of the following factors would increase your acceptance of getting the COVID-19 vaccine when it becomes available?

	Strongly decrease	Decrease	Slightly decrease	No Impact	Slightly Increase	Increase	St in
Your own doctor recommends the vaccine.	○	○	○	○	○	○	
The CDC recommends the vaccine.	○	○	○	○	○	○	
Your state's public health department recommends the vaccine.	○	○	○	○	○	○	
Donald Trump recommends the vaccine.	○	○	○	○	○	○	
Your pastor recommends the vaccine.	○	○	○	○	○	○	
Your coworkers get vaccinated.	○	○	○	○	○	○	
Your friends/family get vaccinated.	○	○	○	○	○	○	
You're only allowed to do certain things, like eat in restaurants, if you're vaccinated.	○	○	○	○	○	○	

What are the main reasons that you would not agree to receive a coronavirus/COVID-19 vaccine, if one was available now? You can choose multiple answers.

I don't think the vaccine would work.

I don't think I need the vaccine.

I have religious objections.

I think it was created too quickly.

I want to wait to be sure it's safe.

I don't trust vaccines overall.

I think it's better to wait for "herd immunity" to naturally develop.

I want to wait to see how effective it is.

I think that political interests are interfering with vaccine development.

I don't trust the scientists developing the vaccine.

Other reason:

I don't trust this new type/technology of vaccine.

Please indicate how much you agree with [believe] the following statements.

	Definitely true	Probably true	Neither true nor false	Probably false	Definitely false
You don't need to wear a mask.	○	○	○	○	○
The Black Lives Matter protests led to increased transmission.	○	○	○	○	○
Wealthy elites intentionally spread the virus to win power and profit.	○	○	○	○	○
Hydroxychloroquine is an effective treatment.	○	○	○	○	○
The new coronavirus was engineered in a lab in China.	○	○	○	○	○
We can safely achieve herd immunity by letting the virus spread through the population.	○	○	○	○	○
Spikes in cases are because of increased testing.	○	○	○	○	○
Any vaccine will be unsafe and a bigger risk than getting COVID-19.	○	○	○	○	○
COVID-19 is no worse than the flu.	○	○	○	○	○
If COVID were more dangerous, I would be more likely to get a vaccine.	○	○	○	○	○

How safe and effective do you think the vaccine against COVID-19 will be in the general population?

Not at all — Completely

0 10 20 30 40 50 60 70 80 90 100

Safety of the vaccine

	Not at all										Completely
	0	10	20	30	40	50	60	70	80	90	100
Effectiveness of the vaccine											

Imagine that a close friend or member of your family got the COVID-19 vaccine before you. How likely would you be to...

	Not at all likely	Slightly likely	Somewhat likely	Pretty likely	Very likely
Spend time with them in person, wearing a mask	O	O	O	O	O
Spend time with them outdoors, wearing a mask	O	O	O	O	O
Spend time with them outdoors, without a mask	O	O	O	O	O
Go over to their house, without a mask	O	O	O	O	O
Share a meal with them in a restaurant, without a mask	O	O	O	O	O

As a health care worker, your opinion on health topics is likely important to people in your life both personally and professionally.

How willing would you be to recommend the COVID-19 vaccine to...

	Extremely likely	Moderately likely	Slightly likely	Neither likely nor unlikely	Slightly unlikely	Moderately unlikely	Extremely unlikely
Family	O	O	O	O	O	O	O
Friends	O	O	O	O	O	O	O
Patients	O	O	O	O	O	O	O
Coworkers	O	O	O	O	O	O	O

What concerns do you have about discussing/recommending COVID-19 vaccination to patients?

COVID Personal Experience

How much has your life been affected by the pandemic?

Not at all	A little	A moderate amount	A lot	A great deal

In what ways has your life been negatively affected by the pandemic?

I lost my job / my hours have been cut down

I have had to homeschool my children

I have had to quit my job to take care of my family at home

My financial situation is worse than before the pandemic

I have tested positive for COVID-19

I have not been able to visit with family

I have experienced more anxiety/stress since the pandemic started

Other

Think about yourself and the people in your life. To the best of your knowledge, who among the following groups has contracted COVID-19?

	Yes	No
Myself	○	○
A family member I live with	○	○
A family member I don't live with	○	○
A close friend	○	○

How likely are you...

	This DEFINITELY won't happen to me										This DEFINITELY will happen to me
	0	10	20	30	40	50	60	70	80	90	100
To get COVID-19 in the near future?											
To get very sick, if you caught COVID-19?											
To die, if you caught COVID-19?											

General Vaccination Questions

Have you gotten a flu shot this season?

Yes

No, but I probably will

No, but I might

No, and I probably won't

No, and I definitely won't

How would you categorize your attitude toward **vaccination in general**?

Extremely against vaccination = -50
Neither anti- nor pro-vaccination = 0
Extremely pro-vaccination = 50

	Extremely against vaccination					Neither anti- nor pro-vaccination					Extremely pro-vaccination
	-50	-40	-30	-20	-10	0	10	20	30	40	50
I am...											

Practice variables

Which of the following best describes you? Please select all that apply.

Physician/mid-level provider

Nurse

Nurse assistant

Pharmacist

Administrator

Indirect patient service provider (e.g., lab tech, food services, environmental services, etc.)

Other (please specify)

As part of your job, do you or will you...

	Yes	No
Play any role, directly or indirectly, in caring for individual patients?	O	O
Come into direct contact with possible COVID-19 patients?	O	O
Administer the COVID-19 vaccine to patients when it's available?	O	O

How large is your hospital?

Under 25 beds

25-49 beds

50-99 beds

100-199 beds

200-299 beds

300-399 beds

300-499 beds

500 beds or more

Demographics

What is your gender?

Female

Male

Non-binary/Genderqueer

Additional category not listed here:

What is your ethnicity? Select all that apply:

American Indian or Alaska Native (for example, Navajo Nation, Blackfeet Tribe, Mayan, Aztec, Nome Eskimo Community)

East Asian (for example, Chinese, Japanese, Korean)

South Asian (for example, Indian, Pakistani, Nepalese)

Southeast Asian (for example, Vietnamese, Thai, Burmese)

Black or African American (for example, Jamaican, Haitian, Nigerian, Ethiopian, Somalian)

Middle Eastern or North African (for example, Lebanese, Iranian, Egyptian, Syrian, Moroccan, Algerian)

Native Hawaiian or Other Pacific Islander (for example, Native Hawaiian, Samoan, Chamorro, Tongan, Fijian, Marshallese)

White (for example, European American, German, Irish, English, Italian, Polish, French)

Hispanic or Latino/Latina/Latinx (for example, Mexican or Mexican American, Puerto Rican, Cuban, Salvadoran, Dominican, Columbian)

Multiracial

An additional category not listed:

What is the highest level of school you have completed or the highest degree you have received?

What is your current annual household income?

In which state/province do you live?

What is your zip code?

Here is a sliding scale on which the political views that people might hold are arranged from extremely liberal (left) to extremely conservative (right). Where would you place yourself on this scale?

	Liberal				Moderate				Conservative		
	0	10	20	30	40	50	60	70	80	90	100
Political Views											

Generally speaking, do you usually think of yourself as a Republican, a Democrat, an Independent, or something else?

Republican

Democrat

Independent

Other (please specify)

No preference

Thank you for participating in our survey! Please click HERE to enter your email address in a separate survey for our prize drawing.

Powered by Qualtrics
